# A New Start

**DOI:** 10.1021/EHP.6c00320

**Published:** 2026-05-05

**Authors:** Joel D Kaufman

**Affiliations:** Departments of Environmental & Occupational Health Sciences, Medicine, and Epidemiology, 7284 University of Washington, Seattle, Washington 98195, United States

The May 2026 issue marks the return of *Environmental Health Perspectives* (*EHP*) in a new publishing home. Now published by ACS Publications, the journal resumes a trajectory that began in 1972 at the National Institute of Environmental Health Sciences. *EHP* did not simply track the emergence of environmental health sciences (EHS); it helped organize and shape the field.

In a closing editorial published by *EHP* at NIEHS, I reviewed the journal’s history and briefly noted the somewhat chaotic forces that led to the end of new submissions in April 2025 and a final issue published in June of last year.^1^ We will return to this history in future editorials, but for now, we can have a fresh start with a new publisher committed to continuing the journal’s legacy. The journal has reopened to submissions, is releasing manuscripts that were accepted but not fully published, and is restoring its archive. Over the next few months, prior *EHP* content will reappear on the new journal site, alongside new manuscripts that are published as they are accepted.

The scope of *EHP* is unchanged, and we anticipate that the bar for publication will remain high. The journal will continue to consider work across the EHS disciplines including toxicology, epidemiology, exposure science, methods, risk assessment, policy, and community-based research—focused on addressing the human health impacts of environmental factors. We aim to highlight all the steps along the translational path in environmental health, from scientific discovery, through careful interdisciplinary investigation, and ultimately to public health action and evaluation.^2^ The expectation, however, is not breadth alone. Papers should generally provide novel evidence or approaches, elucidate mechanisms, strengthen inference, or alter how evidence is used in practice or policy. Rigor and transparency in reporting remain essential. Category names have been updated to match ACS Publications conventions (e.g., Research Articles are now Articles, Seminars are Forums, Research Letters are Letters to the Editor), but the substance of what is considered publishable has not shifted.

Furthering our emphasis on continuity, we are reconstituting the editorial structure. Although we celebrate the retirement of former Deputy Editor Manolis Kogevinas, we welcome back Sara Adar, Dana Barr, B. Paige Lawrence, and Jennifer Peel as Executive Editors. Former Associate Editors are being recruited back to the journal, and additional appointments will follow.

One of the essential elements of *EHP*’s success over the years was the editorial independence that NIEHS leadership granted to the journal. The journal has been able to focus on rigorous science and well-supported scientific perspectives. A succession of NIEHS institute directors, starting with David Rall, made a clear commitment not to interfere with editorial decisions. ACS Publications has affirmed that its governance structure maintains that separation: academic editors retain authority over what is published and how decisions are made. These guarantees are critical as we navigate the journal’s future and to assure our readers and authors that editorial decisions are not influenced by the financial interests of the publisher’s parent society, the American Chemical Society.

The outpouring of support for the return of *EHP* has been a key driver of this relaunch. While this launch is a source of celebration, it is important to acknowledge that many valuable aspects of EHP Publishing at NIEHS have been lost. Most important is the inability to maintain the editorial and management staff and contractors—the people who were primarily responsible for the quality and integrity of the journal. While most have found new positions, the human impact of the journal’s closure at NIEHS is real and represents a loss to the EHS community. There are many other losses as well. The discontinuation of the *Journal of Health and Pollution*, which provided a venue for work from low- and middle-income countries and Indigenous communities, leaves a gap that has yet to be filled.

Please join me in thanking ACS Publications for shepherding *EHP* into its next chapter. I hope that the supportive response from authors, readers, reviewers, and editors will translate into a successful relaunch for the journal. The measure of this relaunch will not be the fact of return. It will be whether the journal reestablishes itself as a trusted venue for reading and publishing consequential research that advances understanding and management of environmental risks to improve human health. That outcome will depend on what is submitted, how it is reviewed, and the standards that are applied. Welcome back to *EHP.*
